# Implementation of Electronic Consent at a Biobank: An Opportunity for Precision Medicine Research

**DOI:** 10.3390/jpm6020017

**Published:** 2016-06-09

**Authors:** Natalie T. Boutin, Kathleen Mathieu, Alison G. Hoffnagle, Nicole L. Allen, Victor M. Castro, Megan Morash, P. Pearl O’Rourke, Elizabeth L. Hohmann, Neil Herring, Lynn Bry, Susan A. Slaugenhaupt, Elizabeth W. Karlson, Scott T. Weiss, Jordan W. Smoller

**Affiliations:** 1 Partners HealthCare Biobank, Partners HealthCare Personalized Medicine, Boston, MA 02139, USA; nboutin@partners.org (N.T.B.); ahoffnagle@mgh.harvard.edu (A.G.H.); nmallen@partners.org (N.L.A.); sweiss@partners.org (S.T.W.); 2 Massachusetts General Hospital, Boston, MA 02114, USA; kmathieu@mgh.harvard.edu (K.M.); ehohmann@mgh.harvard.edu (E.L.H.); sslaugenhaupt@mgh.harvard.edu (S.A.S.); 3 Partners Research Information Systems and Computing, Partners HealthCare Systems, Boston, MA 02115, USA; vcastro@partners.org; 4 Human Research Affairs, Partners HealthCare, Boston, MA 02116, USA; mmorash@partners.org (M.M.); porourke@partners.org (P.P.O.); 5 Department of Pathology, Brigham and Women’s Hospital, Boston, MA 02115, USA; nherring@partners.org (N.H.); lbry@partners.org (L.B.); 6 Department of Neurology and the Center for Human Genetic Research, Boston, MA 02114, USA; 7 Channing Division of Network Medicine, Brigham and Women’s Hospital, Boston, MA 02115, USA; ekarlson@partners.org; 8 Psychiatric and Neurodevelopmental Genetics Unit, Center for Human Genetic Research, Boston, MA 02114, USA

**Keywords:** biobank, electronic consent, precision medicine, informed consent

## Abstract

The purpose of this study is to characterize the potential benefits and challenges of electronic informed consent (eIC) as a strategy for rapidly expanding the reach of large biobanks while reducing costs and potentially enhancing participant engagement. The Partners HealthCare Biobank (Partners Biobank) implemented eIC tools and processes to complement traditional recruitment strategies in June 2014. Since then, the Partners Biobank has rigorously collected and tracked a variety of metrics relating to this novel recruitment method. From June 2014 through January 2016, the Partners Biobank sent email invitations to 184,387 patients at Massachusetts General Hospital and Brigham and Women’s Hospital. During the same time period, 7078 patients provided their consent via eIC. The rate of consent of emailed patients was 3.5%, and the rate of consent of patients who log into the eIC website at Partners Biobank was 30%. Banking of biospecimens linked to electronic health records has become a critical element of genomic research and a foundation for the NIH’s Precision Medicine Initiative (PMI). eIC is a feasible and potentially game-changing strategy for these large research studies that depend on patient recruitment.

## 1. Introduction

Biobanks, which compile, process, and store human biological specimens for future research purposes, have become a key platform for enabling biomedical research [1]. Biobank specimens are often linked to medical health records or supplemented with other data to enable genetic or biomarker studies of a diverse range of clinical phenotypes. These biobanks are excellent resources for the study of genetically complex or rare diseases, as both require large numbers of specimens to perform genetic and biomarker studies [2]. Large biobanks are also a powerful resource for longitudinal studies of disease course and treatment outcomes. A number of large biobanks have been established to meet these needs in the United States and internationally [1]. For example, biobanks that include broad phenotypic data have enabled phenome-wide association studies that can identify novel genetic risk factors and characterize the penetrance and spectrum of their phenotypic effects [3]. In 2015, President Obama announced his intention to launch a Precision Medicine Initiative (PMI) to enhance innovation in biomedical research through the promotion of personalized medicine. Access to biological specimens through the establishment of a biobank is an essential component of this vision [4].

Building a large biobank typically requires obtaining informed consent from a concordantly large number of participants. Traditionally, biobanks have recruited participants through targeted mailings [5], in-person encounters with research assistants in clinical settings, or opt-out methods in healthcare systems [6,7]. Specimens can then be collected during routine clinical draws, as dedicated research draws, or as discarded excess clinical samples.

The Partners HealthCare Biobank (Partners Biobank or Biobank) is a large biospecimen and data repository established to help drive translational biomedical research at Massachusetts General Hospital, Brigham and Women’s Hospital, and other institutions affiliated with Partners HealthCare. The Biobank provides researchers at these institutions with DNA, plasma and serum specimens, genomic data, and survey data that are linked to the longitudinal clinical records of consented subjects. The survey data is collected in a self-reported survey on lifestyle, environment, and family history. The genomic data results from an initiative to genotype 25,000 specimens leveraging the Illumina Multiethnic Beadchip Array (MEGA). Participants provide consent for a broad use of specimens and data as well as for recontact for future studies. The Biobank leverages coded and narrative electronic heath record (EHR) data to create statistically validated phenotypes [8]. The Biobank launched electronic informed consent (eIC) tools and processes in June 2014 as part of its strategy to recruit 75,000 consented subjects by 2018.

Here, we discuss our experience with a large-scale implementation of eIC, including its impact on operations, recruitment, and participant engagement. This experience may inform the development of electronic consent methods envisioned by the Precision Medicine Initiative (PMI) cohort program. The PMI, announced by President Obama in his 2015 State of the Union address, aims to advance the goal of improving health outcomes by using genetic and other individual differences to develop more effective, tailored treatment approaches. The Initiative’s centerpiece, the PMI Cohort Program, will build a national research cohort of one million or more participants and collect biospecimens linked to EHR, mobile health data, and other data sources. Establishing secure, efficient electronic tools for obtaining informed consent will be essential to the success of the program.

### Traditional Approaches to Informed Consent

The Partners Biobank began recruiting patients through traditional recruitment methods in 2010. A typical workflow includes sending a letter to a patient in advance of a clinical visit, then approaching the patient in the waiting room before the visit. A research assistant provides the patient with a high-level overview of the Biobank and conducts the informed consent process. At the Partners Biobank, a software application enables research staff to record whether the patient provides consent, declines to provide consent, or has been contacted without making a decision. In a 20-month period from June 2014 through January 2016, 51% of patients approached through this traditional approach provided consent.

Mailings and in-person enrollment strategies are effective and can enable the recruitment of thousands of participants, but they are time-consuming and expensive to scale in terms of personnel and other resources. At the Partners Biobank, consenting 75,000 participants over five years would be prohibitively expensive.

A 2010 survey of 16 international biobanks found that none successfully recovered their costs [1]. The same study found that population-based biobanks are even more expensive in the long-term when considering the cost of preserving and maintaining tens of thousands of samples. The high costs of recruitment and sample management create incentive for biobanks to find ways to reduce or defray capital and operational costs.

Additionally, recruiting and consenting all patient populations within a large healthcare system requires constant monitoring of recruitment rates, the number of patient visits at specific sites, and individual clinic requirements for approaching their patient populations for consent. These activities require dedicated team leads for the recruitment to ensure a maximum yield of recruitment at the highest yield clinical sites within the hospitals. While research assistants can use scheduling data to determine where to prioritize recruitment efforts, schedules can be imprecise in day-to-day recruitment activities as patients may reschedule their appointments. Another constraint is the lack of adequate space in clinic settings to recruit patients and draw blood.

An alternative approach for building a biobank is the opt-out method, which was employed by the Vanderbilt University Biobank (BioVU) until recently, when a full-consented model was adopted. BioVU originally collected discarded clinical samples from patients by using a short “consent to treatment” form [9]. In this system, the form is signed at each hospital admission or outpatient visit, and the patient can opt-out of participation by checking a box on the form. The sample is linked to a de-identified version of that patient’s electronic medical record. The Vanderbilt Institutional Review Board (IRB) agreed that de-identified samples met the criteria for “nonhuman subjects research” and thus did not require additional informed consent procedures.

While opt-out methods can be highly efficient for building a very large biobank, changes in the National Institute of Health (NIH) policy may make such strategies problematic. Specifically, the NIH Genomic Data Sharing Policy has required all future NIH-funded studies using genomic or phenotypic data for research (beyond a certain sample threshold) to first obtain informed consent from the participant, regardless of whether the subsequent data are de-identified or not [10]. Informed consent is also an essential component of the vision that PMI has laid out. Further, the Notice of Proposed Rule Making (NPRM) to Human Subject Federal Regulations (Common Rule) proposes to expand the definition of human subjects to all biospecimens, regardless of identifiability, and require broad consent for the future use of clinical specimens. This proposed change further emphasizes the importance of consent.

## 2. Materials and Methods

### Electronic Approach to Informed Consent

**Figure 1:**
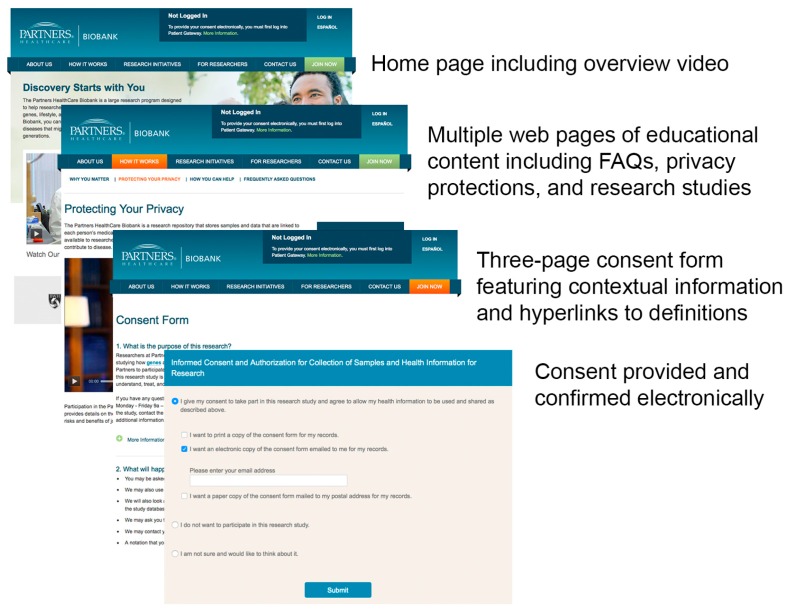
Screenshots from the Biobank eIC website, illustrating key components of the eIC presentation, (Illustration of eIC design).

At Partners HealthCare, eIC is accomplished through a website (https://biobank.partners.org/) that features multimedia content about the Biobank (Figure 1). The core of the informed consent process is an electronic version of the Biobank consent form that is enhanced with contextual information and definitions. After reading each section in the consent form, users may click on an icon to view additional information. Users may also click underlined key words to view definitions. The online format inherent to eIC allows the consent form to be presented in three pages compared to five pages for the print version showing the same content.

The website features content that was designed based on the questions that patients typically ask during the in-person informed consent process. A short overview video on the purpose of the Biobank is featured on the home page and a focused video on patient privacy may be played from another page that focuses exclusively on privacy. The website’s design is adapted for different platforms, such as mobile devices, thanks to responsive design. The website is also entirely translated into Spanish.

In order to provide electronic consent, patients must first log into Patient Gateway, the patient portal at Partners HealthCare, and click on a link to the Biobank website. This is a key feature, as it ensures that each patient’s identity is clearly authenticated in Patient Gateway through the use of a unique user name and password. Per the most common eIC workflow, a patient logs into Patient Gateway after receiving an email invitation from the Biobank. The patient then browses through the educational content on the website, clicks “Join Now” on the top menu, reads the consent form, and has the option to provide consent. The patient then fills out a 10–15-min health information survey relating to lifestyle, environment, and family history, and views instructions on how to provide a blood sample.

In addition to patient authentication, security is an essential component of the eIC strategy at the Partners Biobank. The eIC website makes use of standard web security measures such as secure network connections between client (browser) and server and between the application and enterprise services. Web servers are kept up to date with security and Operating System level patches. Data are stored in an encrypted state. Moreover, the application is periodically subjected to a security assessment by an external application security vendor.

**Figure 2:**
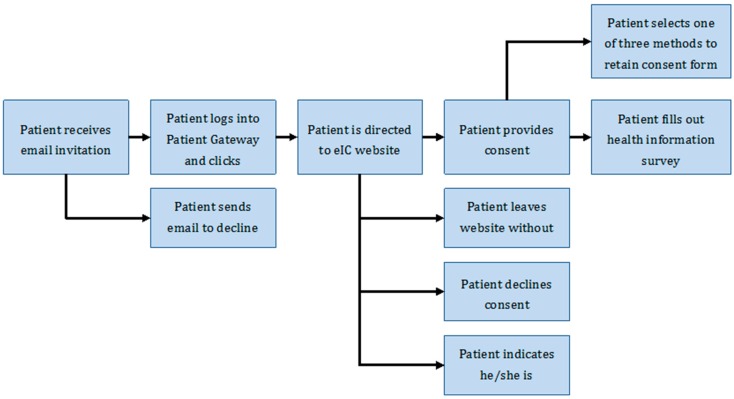
Overview of workflow for eIC at the Partners Biobank.

Subjects provide consent by clicking a radio button that states “I give my consent to take part in this research study and agree to allow my health information to be used and shared as described above”. Selecting this radio button triggers a series of questions to capture how the patient will retain a copy of the consent form. Retaining a copy of the consent form is a key IRB requirement. The three options are to print a copy, to receive an electronic copy by email, or to receive a paper copy by post. Before recording the patient’s consent, the website displays a pop-up window for patients to explicitly confirm their decision to participate in the Biobank. Once that validation is provided, the user is led to the health information survey. A schematic of the overall workflow for eIC is shown in Figure 2.

The conceptualization, design, and implementation of eIC took two years to complete. The IRB was closely involved through the entire process to ensure that this novel method would meet all required standards of recruitment and consent.

Managing a large email campaign is a critical component of the eIC strategy at the Partners Biobank. A critical success factor for any electronic consent strategy is the ability to draw large numbers of people to the eIC tool. The Biobank’s IRB protocol allows for three invitation emails per patient in the first year and a fourth email in the following year. All emails are sent in the 7–14-day window prior to a clinical appointment. The rollout of this email campaign was gradual, starting in July 2014 with a batch of just 20 emails. The number of emails sent each week was increased gradually to ensure that the Biobank staff could properly manage the scale of our operation; by October 2014, first emails were sent to 4000–8000 patients each week. The number of first emails has since stabilized to approximately 1500–2000 each week. A software application was implemented to help manage the Biobank’s email campaign, including the ability to configure the number and frequency of research emails that are sent to the patient population at Partners HealthCare. This application has now been extended to manage email campaigns for other research studies.

## 3. Results

**Figure 3:**
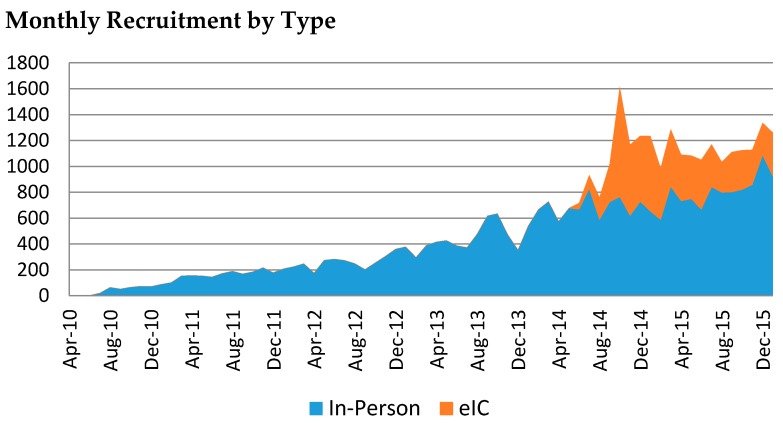
Timeline of the number of participants enrolled until January 2016 by in-person consent (blue) and eIC (red), which began in June 2014.

The website that enables eIC went live in June 2014. During the first 20 months of operation, a total of 7078 patients provided their consent via eIC; 11 patients later withdrew their consent (0.16%). Only 610 (9%) of these patients provided their consent without first receiving an email invitation (referred to as “eIC volunteers”) (Figure 3). The remaining 91% were invited to participate in the Biobank via email.

From July 2014 through January 2016 the Biobank invited every eligible patient with an active Patient Gateway account who had an upcoming clinical appointment on the main campuses of MGH and BWH (*n* = 184,387) to participate in the Biobank. At the end of January 2016, 3.5% of all patients who received an email provided electronic consent (Table 1). Of note, each of the four emails sent to patients during this time period yielded a rate of consent between 1.0% and 1.5%. The Biobank is approved to send a fourth email to patients one year after the third email. Fourth emails started going out in October 2015. To date, the rate of consent for this fourth email is another 1.0% (*n* = 12,881). Thanks to this fourth email, the rate of consent of emailed patients is expected to increase over time. Another metric for evaluating the success of eIC is to measure the consent rate of patients who log into the eIC website. Patients may reach the website as a result of the email invitation, though an unknown proportion may do so independently. For the first 20 months of operation, 30% of patients who were authenticated into the website provided consent. Notably, this is nearly ten times greater than the overall proportion of consenting patients among those who received the email invitation. However, it is lower than the rate of consent for patients approached in person in the clinic (51%).

In order to provide consent, patients must be logged in using their unique Patient Gateway user name and password. Patients may decline participation by responding to the Biobank’s email or by logging into the Biobank website and selecting ‘Decline’ from the home page. Providing patients with an easy way to unsubscribe from the email campaign was a key requirement. Of the patients who receive email invitations, 1.0% decline consent, and most do so by sending an email rather than logging into the website.

Based on consent data for the first 35,997 Biobank participants, we found no significant difference in the age of the participants who consent electronically *versus* those who consent in person. However, those who provide consent electronically were more likely to be female, white, and more highly educated (Table 2).

## 4. Discussion

### 4.1. Requirements and Prerequisites

Establishing eIC entailed a number of challenges including identifying and satisfying regulatory, Information Technology (IT), and clinical requirements. As with all clinical research, approval from the IRB at Partners HealthCare was required. Because eIC was such a new approach to recruitment and consent, the Biobank engaged with the IRB very early on to collaborate on best practices. An important element of the IRB approval for eIC was the determination that the Biobank is a “minimal risk” study and that the Biobank is not targeting any specific population within the healthcare system. The IRB was closely engaged with the Biobank team throughout the concept and design phase of the website, including functional and user interface design, and approved all copies. Any modifications to the website’s copy, functionality, or user interface are submitted for IRB approval.

The Biobank also consulted with institutional legal advisors in the development of eIC. In particular, it was important to assess requirements for use and validity of electronic signatures in documenting informed consent and authorization for use and disclosure of identifiable data. Federal research and privacy regulations provide some guidance but ultimately defer to laws governing electronic signatures in the local jurisdiction, which required analysis.

An essential component for the success of eIC was patient authentication via Patient Gateway, the patient portal at Partners HealthCare. This required approval by a committee that governs clinical initiatives across the healthcare system. While there are other ways to verify patient identity, authentication through the use of a unique username and password is an excellent solution that, in our case, provided the advantage of leveraging the existing infrastructure and processes.

Finally it was important to have the support and approval from clinical leadership across the participating hospitals. Plans for email recruitment and eIC were approved by hospital presidents, the Chief Medical Officers, and the Physician Organization leadership.

### 4.2. Benefits

The greatest benefit of eIC has been a substantial increase in the number of consented Biobank participants. From June 2014 through January 2016, 32% of all Biobank participants provided their consent via eIC, representing 20% of the total Biobank cohort. Moreover, the emailing campaign designed to invite all MGH and BWH patients to participate in the Biobank has enabled outreach to thousands of patients each week, increasing awareness of the Biobank across the healthcare system. This experience in establishing this infrastructure and educational outreach may be instrumental to implement the future human subject biospecimen regulations that may be required in the near future.

In addition, eIC has the potential to enhance the informed consent experience for participants. The use of interactive multimedia has been shown to increase patient understanding and confidence in the understanding of informed consent documents [11]. Of note, while the Biobank did run focus groups and surveys as part of a pilot assessment of its eIC solution in July and August 2014, we have not yet assessed whether eIC is preferred by patients compared to in-person recruitment.

Finally, eIC can provide patients more time for decision-making [12]. Patients can explore the Partners Biobank website at their own speed and may also reach out to Biobank staff with additional questions. For some patients, this flexibility may facilitate participation. Indeed, recent evidence suggests that time constraints may play a substantial role in the decisions to consent for participation in a biobank. In a study from the Mayo Clinic [13], 62% of 1600 adults who were mailed biobank recruitment packets did not respond. Follow-up interviews with non-responders indicated that the vast majority (73%) claimed that they were too busy to comply with the participation requirements, including reading the consent form and completing the survey [13]. Being able to review consent materials at one’s own pace may help overcome this issue. Eligible patients can explore the website to learn about the study whenever it is most convenient for them, and provide consent within the timeframe that works for them.

Every Biobank participant is asked to complete an electronic health information survey that assesses demographics and health behaviors (education, occupation, height, weight, smoking, alcohol intake, sun sensitivity, exercise, sleep, female reproductive history) and family medical history. The rate of completion of this survey for patients who consent electronically is 82%. In comparison, the rate of completion of this survey for patients who consent in person is 39%. Conclusively, eIC is a robust method for ensuring the capture of survey data as part of the informed consent process.

There are also less quantifiable ways in which eIC has affected recruitment and consent. For example, the email campaign that was rolled out in conjunction with the eIC website has greatly increased awareness of the Biobank across the healthcare system—nearly 185,000 patients received an email about the Biobank in the first 20 months. We have found that some patients who receive the email invitation browse the materials online and then wait until their scheduled appointment to discuss participating with a research assistant. This allows assistants to spend less of their limited time explaining the project to patients, and more time answering specific questions, or walking them through the consent process.

By enhancing awareness of the Biobank, in-person recruitment is also facilitated because patients are generally more informed about the project. Additionally, to the extent that eIC substitutes for in-person consent, there is less need for research staff to occupy clinical space; this reduces the risk that recruitment may interfere with clinical workflow. The Partners Biobank has established a dedicated phlebotomy space for patients that have enrolled electronically, bypassing the need to use clinical space.

### 4.3. Challenges and Limitations

While eIC is an efficient recruitment channel, it does present some challenges and limitations (see Table 3). A significant limitation is the dependency of eIC on the available pool of participants whose identity can be electronically validated through the use of a unique username and password. At Partners HealthCare, the pool of potential participants is determined by the patient portal’s user base. When we went live with eIC, this user base was approximately 475,000 MGH and BWH patients, a fraction of the total number of patients seen at these hospitals. On the other hand, the user base of the patient portal is constantly growing, which translates into a constant flow of new potential participants.

A second limitation of our implementation is the dependency on electronic tools, resources, and policies to manage a large-scale email campaign. The number of patients who provide their consent via eIC fluctuates based on the number of email invitations that are sent out each week. Managing such an operation required tools that did not exist at Partners HealthCare. The Biobank built an IT application to manage email lists based on scheduling data, Biobank status, age and vital status, the existence of a Patient Gateway account, and other criteria. This IT application also features business logic to manage the number and frequency of emails that patients may receive. It has now been extended to manage email campaigns for other research studies. Moreover, the Biobank’s experience with emailing large numbers of patients for research has helped inform the development of policies, tools, and best-practices for managing research emails across the healthcare system.

A third limitation of eIC is that the diversity of patients who enroll through this mechanism depends on the community use of computer technology. For example, at the Partners Biobank, we have noted that the patient population enrolling electronically has been less diverse in terms of race and education level compared with those enrolling during in-person interactions with research staff. However, we note that there were no differences in the age distribution of eIC participants, suggesting that age has not been a barrier to acceptance. 

**Figure 4:**
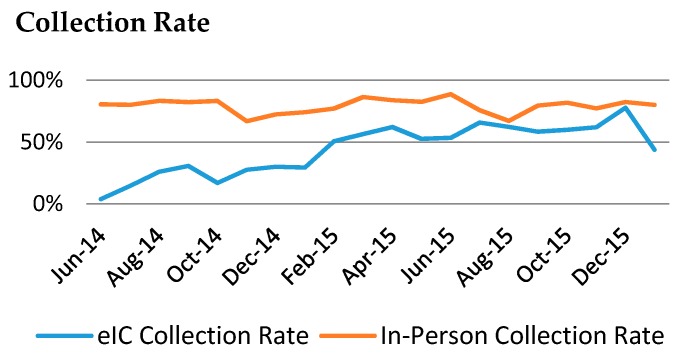
Sample collection rate by in-person consent (red) and eIC (blue) from 1 June 2014 through December 2015.

Finally, because eIC uncouples informed consent from phlebotomy, it requires asynchronous processes to obtain the patient samples, which result in lower collection rates than in-person consent. In early stages of eIC, only 22% of patients who consented electronically followed-up with the Biobank team to provide a dedicated research draw (Figure 4). However, the capacity to include the collection of clinical discards from multiple clinical laboratories through the Crimson system [14,15] helped achieve an average collection rate of 43% by the end of January 2016. This rate for eIC participants has generally been increasing month by month as patients come into the hospitals and have blood drawn for clinical testing. Adding the capacity to include dedicated research tubes in these clinical draws will further improve collections of dedicated samples, a challenge that can be addressed with IT solutions that interface with the clinical phlebotomy and clinical laboratory information systems.

Finally, implementing eIC requires support staff and tools to address patients’ questions and concerns. At Partners HealthCare, for every 1000 patients who receive the first Biobank email invitation, the Biobank receives 20 emails or phone calls with questions (2.0%); responding to these entails several hours each week on the part of the Biobank staff. Moreover, a help desk IT solution is required to manage patient emails, voicemails, and phone calls. On average, the Biobank receives 185 such communications each month. Moreover, implementing eIC also requires IT staff. Whether the eIC tool is custom built or implemented using an off-the-shelf software package, it will require regular maintenance, including software updates, server upgrades, and functional changes.

Of note, while the Partners Biobank considers eIC a successful strategy, it will take subsequent years of evaluation of the science and clinical activities that have been supported to quantify the magnitude of this success. Given the lack of widespread use of eIC in other biobanks, there are few metrics by which to benchmark our performance in terms of outreach, patient engagement, and consent rate. Over time, as other institutions launch large eIC initiatives, some better benchmarks will be established to qualify success.

## 5. Conclusions

Over the past five years, Partners HealthCare has established a Biobank of patient samples linked to longitudinal electronic health records. Here, we’ve outlined our experience in developing this Biobank and implementing a novel recruitment method.

In our experience, eIC provides a useful supplement to in-person recruitment methods. The benefits of eIC have included a vast expansion in patient outreach, increased enrollment into the Biobank, and a novel opportunity for patients to engage in a thorough informed consent process. At the same time, successful implementation of an eIC strategy entails challenges in terms of dependence on a robust infrastructure to validate patient identity, tools to manage large-scale outreach and support, and dependence on patient engagement with technology. Of course, the cost to develop the infrastructure is not insignificant. Nevertheless, we have demonstrated that eIC is a feasible and potentially game-changing strategy for large research studies that depend on patient recruitment. As recommended by the PMI, we hope that our experience at Partners HealthCare will help inform best practices and benchmarks for electronic informed consent.

## Figures and Tables

**Table 1:** Results of the email campaign (June 2014–January 2016).

Rate of Consent by Emailed Patient	Patients	Consent Rate
First email	184,387	1.5%
Second email	163,238	1.4%
Third email	114,327	1.1%
Fourth email	12,881	1.0%
Any email	184,387	3.5%
**Rate of Consent by Website Visitors**	**Visitor**	**Consent Rate**
Logged-in visitors (excluding those who consented in person)	23,562	30%

**Table 2:** Comparison of in-person and eIC demographics (Jun 2014 to Jan 2016).

Title	In-Person	eIC	Total	*p*-Value
**N**	28,930	7067	35,997	
**Age**				0.302 ^1^
Average age	56.5	56.7	56.5	
**Gender (%)**				<0.0001 ^2^
Female	57	60	58	
Male	43	40	42	
**Race (%)**				<0.0001 ^2^
Asian	2	2	2	
Black	7	1	6	
Hispanic	5	1	4	
White	81	92	83	
Other	2	1	2	
Unknown	4	3	4	
**Education (%)**				<0.0001 ^2^
8th grade or less	2	0	1	
Some high-school	3	0	2	
High school/GED	18	6	16	
Some college	6	4	6	
College	51	72	55	
Graduate school	2	4	3	
Unknown	18	14	17	

^1^ Student’s *t*-test *p*-value; ^2^ Chi-square *p*-value comparison of all categories.

**Table 3:** Pros and cons of in-person *vs.* electronic informed consent.

Format	Pros	Cons
In-Person Consent	Effective and well-established method	Consent rate limited by personnel availability Requires space in clinical setting High operational cost due to staffing requirements
Electronic Informed Consent	Recruitment engine with low operational cost after initial capital investment Recruitment capacity beyond that feasible with in-person approach Potential to enhance informed consent experience and provides prospective enrollees more time for decision-marking	Requires internet access for prospective enrollees Need solutions for completing biospecimen collection, as consent process is disassociated from collection Requires investment in IT infrastructure for consent platform and secure authentication Resources and policies needed to manage large-scale email campaign
